# Seasonal patterns of bison diet across climate gradients in North America

**DOI:** 10.1038/s41598-021-86260-9

**Published:** 2021-03-25

**Authors:** Joseph M. Craine

**Affiliations:** Jonah Ventures, 5485 Conestoga Ct #210, Boulder, CO 80301 USA

**Keywords:** Grassland ecology, Food webs

## Abstract

North American plains bison (*Bison bison*) have been reintroduced across their former range, yet we know too little about their current diet to understand what drove their past migrations as well as observed continental-scale variation in weight gain and reproduction. In order to better understand the seasonal diets of bison at the continental scale, bison fecal material was collected monthly from April to September in 2019 across 45 sites throughout the conterminous United States. Fecal material was analyzed for dietary quality using near infrared spectroscopy and dietary composition with DNA metabarcoding. As observed in previous research, dietary quality peaked in June and was on average greatest for sites with cold, wet climates. Yet, in April, dietary quality was highest in warmer regions, likely reflecting earlier phenology of plants in southern than northern regions. Independent of climate and season, bison that consumed more warm-season grasses had lower dietary protein concentrations. Interpreting the relative abundance of sequences from different plant species as the relative intake of protein from those species, only 38% of bison protein intake came from grasses. An equal amount of dietary protein came from legumes (38%) and 22% from non-leguminous forbs. Seasonal shifts in bison diet were also clear, in part, following the phenology of functional groups. For example, cool-season grass protein intake was highest in May, while legume protein intake was highest in August. Comparing data taken in June and September 2018 in a previous study with corresponding data in 2019, on average, June [CP] was 20% higher in 2019 than 2018, while September [CP] did not differ between years. Dietary functional group composition was generally similar in amounts and relationships with climate between years, yet in September 2019, legumes contributed 20% more protein and warm-season grasses 14% less than in September 2018. In all, this research demonstrates that bison consistently rely on eudicots for protein with the functional group composition of their diet in some ways consistent across space and time, but also spatially and temporally variable. The early-season inversion of plant quality gradients would have been a strong driver of migratory behavior for large numbers of bison optimizing protein intake. As most bison currently experience protein deficiency, optimizing protein intake under current non-migratory conditions will require increasing the relative abundance of high-protein species such as N_2_-fixing species.

## Introduction

Nutrition is one of the most important keys to understanding the ecology of North American plains bison (*Bison bison*)^1–4^. Many factors such as mineral concentrations and secondary compounds affect grazer nutrition, but protein and energy concentrations of the plants bison eat determine their performance and reproduction, regulate their fine-scale movements as well as long migrations, and structure their interaction with vegetation^3,5,6^.

Some of the distal determinants and consequences of variation of bison nutrition have begun to be quantified. For example, at the continental scale, bison weight gain is greater in cool, wet climates than in hot, dry climates^7^. 6.5-year-old male bison in Ordway Prairie, South Dakota were 260 kg heavier than those in the hotter and drier Wichita Mountains, Oklahoma^7^. This implied that plant protein concentrations aggregated over the year are highest in cool, wet climates. This assertion was subsequently supported by quantifying cattle and bison dietary quality along these climatic gradients^4,8–10^.

Complementary to geographic patterns of bison nutrition, interannual studies of bison performance demonstrated that weight gain and reproduction varies among years^11^, with reproduction dependent on the mother’s mass^12^. For example, in Kansas, a female bison that weighed 320 kg in the fall would have an approximately 20% probability or reproduction the next spring, while one that weighed 520 kg would have > 80% probability. In part, the interannual variation in bison performance was tied to variation in timing and amounts of precipitation, but not in easily predictable ways^11^. For example, in Kansas, years with greater precipitation did not necessarily lead to greater weight gain. Greater precipitation early in the summer led to less weight gain, while precipitation later in the summer lead to greater weight gain. Bison dietary [CP] varies over the year and tends to peak in early summer^4,11^, which is synchronous with period of greatest nutritional demands for lactating bison.

One of the last links in linking geographic or temporal patterns in bison performance with nutrition is to understand the plant species composition of bison diet. Variation in dietary quality is driven by the nutritional quality of individual plant species, but also the relative intake of different plant species. Bison were once thought to consume predominantly grasses^13–17^, but recent studies that have quantified bison diet have shown that it to be much broader. For example, in Oklahoma, about half the seeds found in bison fecal material were from eudicots^18^. In Yellowstone National Park and Alaska, bison regularly browse woody species^19,20^, something also observed for the closely related European bison^21,22^. DNA metabarcoding of plains bison fecal material in Kansas, found that only 39% of the protein intake of bison were from grasses^4^. Even at the northernmost edge of range (Manitoba), less than half of protein bison consumed was from graminoids^23^. In order to begin to assemble continental patterns of bison diet, 50 bison herds were sampled in June and September 2018 across the United States^10^. In that study, forbs and legumes contributed over half the protein to bison diet across their range.

Despite these findings and all that has been learned about geographic and temporal patterns of bison performance, dietary quality, and dietary composition, there is still uncertainty about a number of links among these factors. For example, the geographic patterns of bison weight gain suggest that in aggregate bison nutritional quality over the year is greater in cool, wet climates. Despite the 2018 geographic survey of bison dietary quality, it is still unknown whether the greater dietary quality in cold, wet climates observed in June also held earlier in the season. As a general rule, spring onset east of the Rockies Mountains should occur approximately 4 days later per degree of latitude northward^24^. This would equate to a difference of roughly 52 days between the North Dakota-South Dakota border and central Texas. As such, it is possible that April and May dietary quality could be higher in warmer than cooler climates. It is also unknown what types of plant species bison are relying on early in the season. Similarly, the 2018 project provided no data on dietary quality and composition patterns midsummer, when temperatures are the hottest and nutritional stress is still high from lactation and mating. Regarding interannual patterns, despite the multiple years of data on bison diet for bison in Kansas, we have little understanding of interannual variation of dietary quality and composition for bison across their range. It is unknown whether regional variation in weather could promote some species over others in bison diet, which would potentially explain variation in dietary quality and performance.

Given this background, in order to better understand the seasonal and interannual patterns of bison diet, we collected bison fecal material monthly (April–September) from 45 herds across the US. Fecal material was analyzed with near infrared spectroscopy (NIRS) to quantify dietary crude protein concentrations ([CP]) and digestible organic matter concentrations ([DOM])^25^. The ratio of [DOM] to [CP] is considered an index of energy to protein limitation with ratios above 7 representing strong protein limitation and below 4 strong energy limitation^10,25^. Fecal material was also analyzed for dietary composition with metabarcoding from Next Generation Sequencing^26^. With these data, we sought to (1) understand seasonal patterns of dietary quality and dietary composition, (2) examine how these patterns vary across climate gradients across the six months, and (3) begin to understand interannual variation in dietary quality and composition across the bison’ range with comparisons between the current set of samples and those taken in June and September 2018 across the same climate gradients.

## Methods

### Sample collection

**Figure 1:**
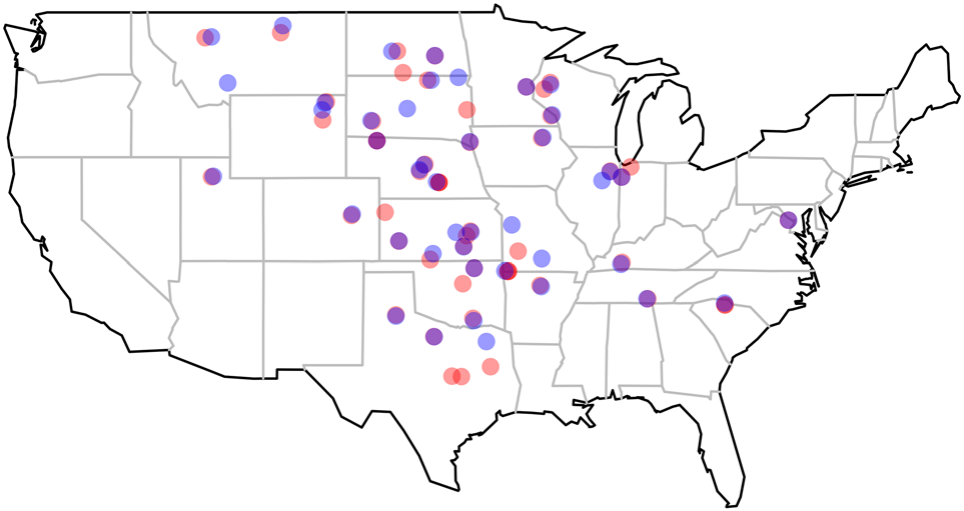
Map of sampling locations for herds sampled in 2018 (red) and 2019 (blue). Colors are semi-transparent such that herds sampled in both years appear purple. Map generated with maps package in R 3.5.2.

The goal of selecting bison herds for the study was to include herds where bison grazed on grasslands representative of a given region without any supplementation of protein or energy. To recruit managers of bison herds for the study, participants of the 2018 study were contacted and emails were sent out to additional members of the National Bison Association requesting their participation. Each registered participant was sent a fecal collection kit, which included a six sampling cups, instructions on sampling, a cooler, gloves, disposable spoons, and an icepack. Participants were instructed to combine small amounts of fresh bison fecal samples from each of 10 fecal pats the first week of each month from April to September. Monthly samples were then frozen and stored until all samples were collected and then sent in to the Grazingland Animal Nutrition Lab (GANLab) at Texas A&M. In all, a total of 260 samples were received as some sites did not collect samples every month. Data for 2 sites from CA were excluded as they fell outside of the continental climate envelope we were investigating. Another site was excluded having had supplemented their animals with protein. A total of 248 samples remained in the data set after exclusion (Fig. 1).

### Dietary quality

Samples received at GANLab were subsequently dried at 60 °C, ground in a Udy mill to pass a 1-mm screen, and analyzed using Near Infrared Reflectance Spectroscopy (NIRS) to assess dietary quality parameters that included crude protein concentrations [CP] and digestible organic matter concentrations [DOM]. Spectra (400–2500 nm) were collected on a Foss NIRS 6500 scanning monochrometer with spinning cup attachment. Calibration curves were derived from NIRS spectra of cattle fecal material and directly measured forage quality^27^ and applied to bison here as previously^4,10,28^.

### Dietary composition

After dietary quality was assessed, dried samples were sent to the Jonah Ventures laboratory in Boulder, Colorado for DNA metabarcoding using the c-h primers of the trnL intron in plant chloroplast^4,29^. Genomic DNA from samples was extracted using the MoBio PowerSoil htp-96 well Isolation Kit (Cat#12,955–4) according to the manufacturer’s protocol. Genomic DNA was eluted into 100 µl and frozen at − 20 °C. Each 25 µL PCR reaction was mixed according to the Promega PCR Master Mix specifications (Promega catalog # M5133, Madison, WI) which included 0.4 µM of each primer and 1 µl of gDNA. Both forward and reverse primers also contained a 5′ adaptor sequence to allow for subsequent indexing and Illumina sequencing. DNA was PCR amplified using the following conditions: initial denaturation at 94 °C for 3 min, followed by 40 cycles of 30 s at 94 °C, 30 s at 55 °C, and 1 min at 72 °C, and a final elongation at 72° C for 10 min. Amplicons were then cleaned by incubating amplicons with Exo1/SAP for 30 min at 37 °C following by inactivation at 95 °C for 5 min and stored at − 20 °C. A second round of PCR was performed to give each sample a unique 12-nucleotide index sequence on the forward read. The indexing PCR included Promega Master mix, 0.5 µM of each primer and 2 µl of template DNA (cleaned amplicon from the first PCR reaction) and consisted of an initial denaturation of 95 °C for 3 min followed by 8 cycles of 95 °C for 30 s, 55 °C for 30 s and 72 °C for 30 s. 5 µl of indexing PCR product of each sample were visualized on a 2% agarose gel to ensure the success of the barcoding PCR. Final indexed amplicons from each sample were cleaned and normalized using SequalPrep Normalization Plates (Life Technologies, Carlsbad, CA). 25 µl of PCR amplicon is purified and normalized using the Life Technologies SequalPrep Normalization kit (cat#A10510-01) according to the manufacturer’s protocol. Samples are then pooled together by adding 5 µl of each normalized sample to the pool. Sequencing occurred on an Illumina MiSeq (San Diego, CA) running the 2 × 150 bp chemistry with a v2 300-cycle kit.

For bioinformatic processing, sequencing success and read quality was verified using FastQC v0.11.8, and reads were demultiplexed by using Illumina-utils v2.6 (iu-demultiplex) using default settings. Sequences of each sample were then merged using the -fastq_mergepairs option in Usearch v11.0.667^30^. The forward primer (5′-CGAAATCGGTAGACGCTACG-3′) and reverse primer (5′-CCATTGAGTCTCTGCACCTATC-3′) were removed using Cutadapt v1.18^31^. Cutadapt is also used to discard sequences with length below 108 bp. Expected error filtering as implemented in Usearch is then used to discard low quality reads (max_ee = 0.5)^32^. Instead of OTU clustering, reads affected by sequencing and PCR errors are then removed using the unoise3 algorithm with an alpha value of 5^33^. This denoising is applied to each individual sample, and Exact Sequence Variants (ESV) compiled in an ESV table including sequences and read counts for each sample. Taxonomy is assigned to each ESV by mapping them against a GenBank reference data^34^ as well as Jonah Ventures voucher sequences records, using usearch_global with –maxaccepts 0 and –maxrejects 0 to ensure mapping accuracy. Consensus taxonomy is generated from the hit tables, by first considering 100% matches, and then going down in 1% steps until hits are present for each ESV. For final statistical analyses, the top 200 ESVs across all sites were used for analyses. The top 200 ESVs represent 89.89% of all reads and no single ESV had less than 10% of the reads for any one sample. Relative abundances for a sample were standardized so the sum of all reads of the top 200 ESVs was equaled 100%. Each ESV was assigned a representative genus if species from multiple genera matched the ESV.

A functional group assignment for each ESV was then made based on the taxonomy assignment of the ESV. Functional group classifications included: cool-season graminoids (grasses with C_3_ photosynthesis as well as *Carex* and *Equisetum* species), warm-season grasses (all C_4_ grasses), legumes (Fabaceae species), herbaceous eudicots, i.e. forbs, and woody eudicots.

### Climate data

Site location was used to obtain 30-year climate normal (1981–2010) from the Oregon State University PRISM Climate Group data explorer database (PRISM Climate Group).

### Statistical analyses

All statistical analyses and figure preparations were conducted in R v. 3.5.2^35^. Beyond standard univariate analyses, a standard least squares model was used to understand the relationship between diet quality and climate overall and for each month. In the models, [CP], [DOM], [DOM]: [CP] were predicted with fixed effects of MAT, MAP, the identity of the month, and all pairwise interactions. To understand whether functional group composition affected dietary quality independent of climate and season, another set of identical models were run but each also included the relative abundance of one of the functional groups. The only functional group that was significant was warm-season grasses and the results of the model including warm-season grasses are reported. In addition to dietary quality, the climate and seasonal influences on functional group composition were tested with models that had the relative abundance of individual functional groups as the response and predictors of MAT, MAP, the identity of the month, and all pairwise interactions. As in no case was the interaction between one of the climate variables and month identity significant, these interactions were removed from the final model. To compare whether dietary quality and composition values in 2018 and 2019 differed, linear models were run for each parameter for each month (June and September) that included just the identity of the year. To test whether relationships between parameters and site climate differed between the years additional linear models were run that included MAP, MAT, identity of the year, the identity of the month, and interactions between 1) MAP and MAT, 2) year and MAT, 3) year and MAP, and 4) year and month identity. Maps of CP and DOM for each month in 2019 and functional composition of diet for June, 2019 were generated utilizing the *raster* package for reclassifying and masking pixels. Forested areas were masked from the map based on data from the Commission for Environmental Cooperation, http://www.cec.org.

## Results

### General patterns of dietary quality and composition

Averaged across the months, site-level [CP] ranged from 62.6 to 147.3 mg g^−1^ and averaged 96.7 ± 3.3 mg g^−1^. [DOM] ranged from 571.3 to 643.6 mg g^−1^ and averaged 605.8 ± 2.4 mg g^−1^ while [DOM]: [CP] ranged from 4.4 to 10.3 with an average of 6.9 ± 0.2.

**Figure 2:**
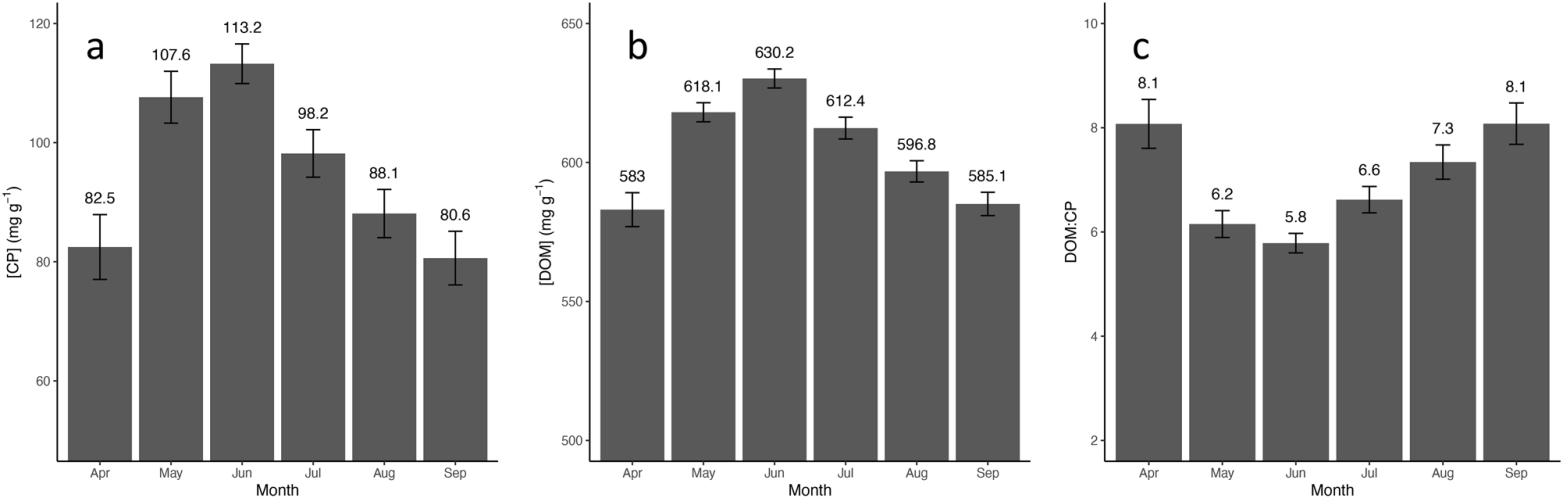
2019 monthly dietary quality metrics averaged (± S.E.) across all sites. Shown are (**a**) crude protein concentrations ([CP]), (**b**) digestible organic matter concentrations ([DOM]), and (**c**) the ratio between [DOM] and [CP].

Examining seasonal patterns averaged across all sites, [CP] averaged 82.5 ± 5.4 mg g^−1^ in April, peaked at 113.2 ± 3.3 mg g^−1^ in June, and declined to 80.6 ± 4.5 mg g^−1^ in September (Supplementary Table S1, Fig. 2). Similarly, [DOM] averaged 583.0 ± 6.1 mg g^−1^ in April, peaked at 630.2 ± 3.4 mg g^−1^ in June, and declined to 585.1 ± 4.2 mg g^−1^ by September (Supplementary Table S1, Fig. 2). [DOM]: [CP] averaged 8.1 ± 0.5 in April, declined to 5.8 ± 0.2 in June, and increased to 8.1 ± 0.4 by September (Supplementary Table S1, Fig. 2).

Examining dietary functional group composition across sites, on average 38.2 ± 2.6% of dietary protein intake came from grasses, ranging from 12.7 to 82.2% across sites. Examining patterns for the two grass functional groups, 26.5 ± 2.9% (1.3–82.0%) of protein intake was derived from cool-season (C_3_) graminoids and 11.7 ± 1.6% (0–36.7%) came from warm-season (C_4_) grasses. Of the Eudicots, protein intake from legumes averaged 37.8 ± 2.8% (1.3–70.2%), from non-leguminous forbs averaged 21.5 ± 2.0% (0.2–68.0%), and 2.5 ± 0.8% (0–31.6%) from woody species.

**Figure 3:**
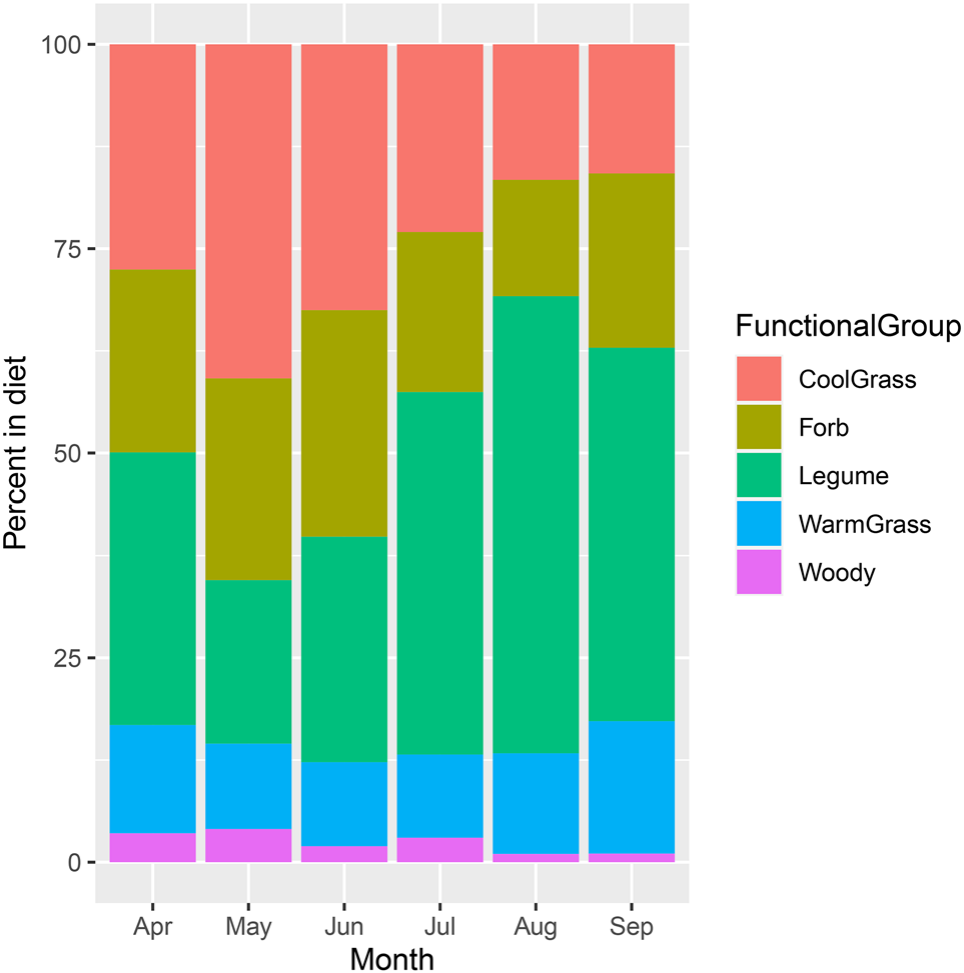
Average contributions of different functional groups to 2019 dietary protein intake for bison across sites for each month from April to September. Data based on the 200 most abundant Exact Sequence Variants (ESVs), which were assigned a consensus taxonomy and then a function classification. Cool-season graminoids includes C_3_ grasses, sedges, and *Equisetum*. Warm-season grass was derived exclusively from grasses. Legumes only included species from Fabaceae.

Examining monthly patterns across sites, C_3_ grass protein intake was highest in May (40.8 ± 5.0%) and lowest in September (15.8 ± 2.8%) while C_4_ grass protein intake peaked in September (16.2 ± 2.9%) and was lowest in July (10.2 ± 2.4%) (Supplementary Table S2, Fig. 3). Legume protein intake was highest in August at 55.8 ± 5.3% and was lowest in May (20.0 ± 4.0%) (Supplementary Table S2, Fig. 3). Forb protein intake peaked in June at 27.6 ± 3.5% and was lowest in August (14.2 ± 2.8%) (Supplementary Table S2, Fig. 3).

### Climate and dietary quality

**Figure 4:**
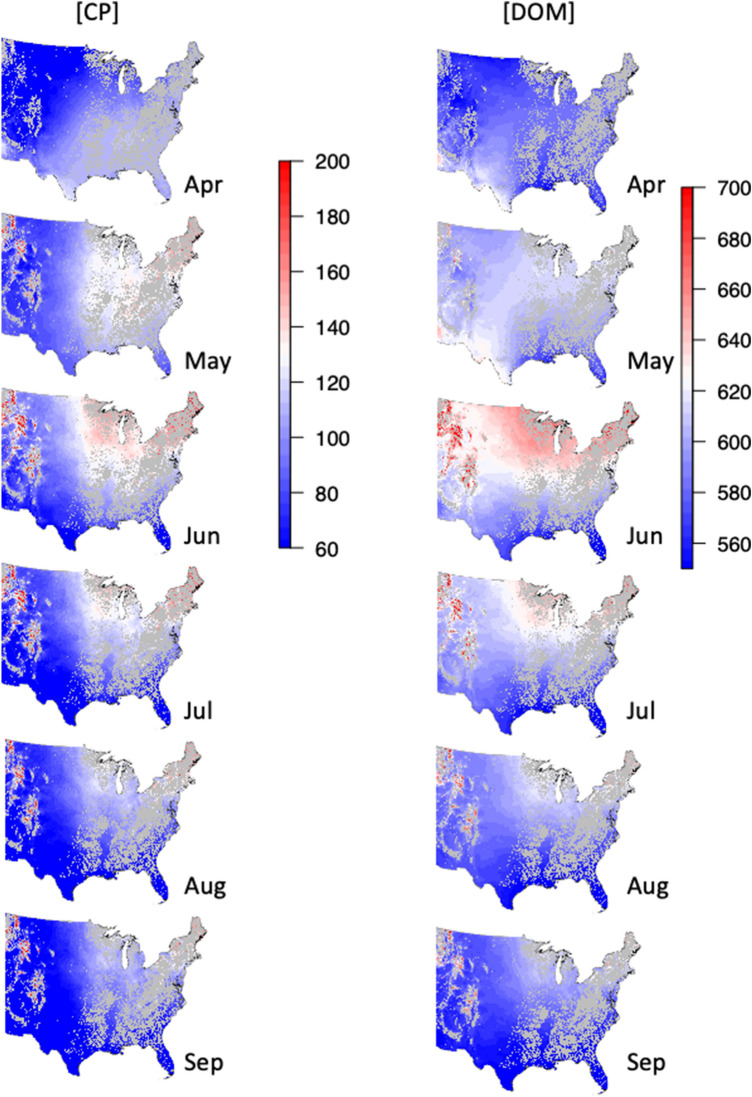
Maps of dietary crude protein and digestible organic matter concentrations for non-forested areas. Maps are modeled based on Supplementary Table S3. Units are mg g^−1^. Maps generated utilizing the *raster* package for reclassifying and masking pixels in R 3.5.2.

Examining patterns of [CP] across sites, [CP] was highest in cool, wet climates (Fig. 4, Supplementary Table S3). For example, bison in June at a site with 1200 mm of rain and 6 °C MAT would have [CP] of 186.3 mg g^−1^. Bison in June at a site with 400 mm of rain and 18 °C MAT would have [CP] of just 67.8 mg g^−1^. There was also a statistical interaction between the identity of the month and MAT on [CP] (Fig. 4, Supplementary Table S3). In April, [CP] tended to increase with increasing MAT (2.51 ± 1.46 mg g^−1^ °C^−1^), as warm-climate sites tended to have higher [CP] than cold-climate sites (Fig. 4, Supplementary Table S3). By May, colder sites tended to have higher [CP] (− 2.01 ± 1.81 mg g^−1^ °C^−1^), which became stronger by June (− 5.34 ± 1.83 mg g^−1^ °C^−1^) and lasted through September (Fig. 4, Supplementary Table S3). Examining patterns of functional group composition on top of climate relationships, [CP] was lower when greater amounts of warm-season grasses were in the diet, independent of climate and season (Supplementary Table S4).

Like [CP], [DOM] was also highest in cool, wet climates (Fig. 4, Supplementary Table S3). For example, bison at a site with 1200 mm of rain and 6 °C MAT in June would have [CP] of 670.4 mg g^−1^ as opposed to bison at a site with 400 mm of rain and 18 °C MAT in June, which would have [CP] of just 580.4 mg g^−1^. In April, bison in warm-climate sites tended to consume a diet that was higher in [DOM] than bison in colder-climate sites (2.04 ± 1.73 mg g^−1^ °C^−1^) (Fig. 4, Supplementary Table S3). By May, [DOM] was invariant across MAT gradients (− 0.08 ± 1.98 mg g^−1^ °C^−1^) and by June, cold-climate sites had higher [DOM] (5.51 ± 2.00 mg g^−1^ °C^−1^) (Fig. 4, Supplementary Table S3).

Examining patterns of DOM:CP reveals the seasonal and spatial patterns of protein limitation. In general, bison in cool, wet climates had the lowest [DOM]: [CP] (Supplementary Table S3). Yet, the statistical interaction between MAT and month shows that in April, bison in cold climates were more protein limited than in warm climates (− 0.16 ± 0.11 °C^−1^) (Supplementary Table S3). By May, [DOM]: [CP] tended to become higher in warm climates, with the gradient becoming strongest by August (0.61 ± 0.15 °C^−1^) (Supplementary Table S3).

### Climate and dietary composition

**Figure 5:**
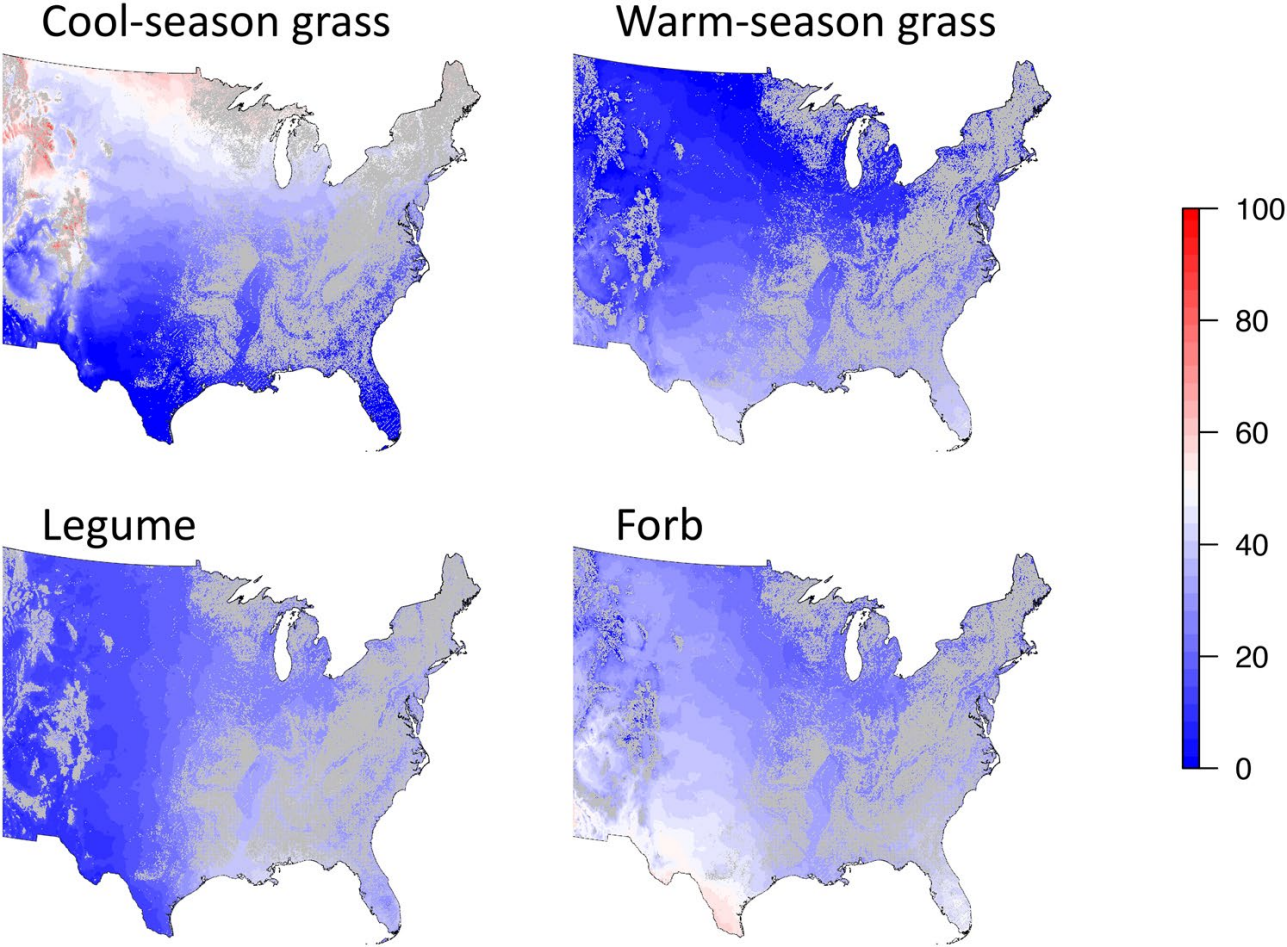
Maps of functional group composition of bison diet (excluding woody plants, which had low abundance and little pattern with climate) for non-forested areas. As there were no interactions between either MAT or MAP and the identity of the month, only maps for June are shown here. See Fig. 2 for how functional group composition of the diet changes over the six six months. Maps generated utilizing the *raster* package for reclassifying and masking pixels in R 3.5.2.

Examining cross-site relationships between the relative amounts of different functional groups in diet and the predictors of climate and season, the proportion of cool-season grass in the diet was highest in cool sites (decreasing at a rate of 3.8 ± 0.5% °C^−1^), with no significant influence of MAP (*P* = 0.16) (Fig. 5, Supplementary Table S5). The proportion of cool-season grass in the diet varied among months, but there was no difference among months in the relationship with MAT (*P* = 0.54). In contrast, the proportion of warm-season grass in diet was greatest in hot, dry regions (Fig. 5, Supplementary Table S5). For example, bison at a site with 400 mm of rain and 18 °C MAT would have 42.9% of their dietary protein from warm-season grasses as opposed to a site with 1200 mm of rain and 6 °C MAT, where warm-season grasses would provide 0% of their dietary protein.

Among Eudicots, there was no seasonal variation in the percentage of forbs in the diet, but the percentage of dietary protein from forbs was highest in hot, dry regions (Fig. 5, Supplementary Table S5). For example, bison at a site with 400 mm of rain and 18 °C MAT in June would have 54.2% of their dietary protein from forbs as opposed to a site with 1200 mm of rain and 6 °C MAT in June, where it would be just 9.6%. Legumes had strong seasonal variation in its dietary contribution, but little variation with climate, increasing at a rate of 2.0 ± 0.8% per 100 mm MAP (*P* = 0.02) (Fig. 5, Supplementary Table S5).

### Comparison with 2018 data

Comparing [CP] between years, June [CP] was higher on average in 2019 than 2018 (113.2 ± 3.3 vs. 93.6 ± 3.2 mg g^−1^; *P* < 0.001). In contrast, September [CP] was not different between the two years (80.6 ± 4.5 mg g^−1^ in 2019 vs. 80.6 ± 4.5 mg g^−1^ in 2018; *P* = 0.98). Like [CP], June [DOM] was higher in 2019 than 2018 (630.2 ± 3.4 mg g^−1^ vs. 617.6 ± 3.7 mg g^−1^; *P* = 0.01) while September [DOM] did not differ between years (585.1 ± 4.2 mg g^−1^ in 2019 vs. 591.7 + 4.4 mg g^−1^ in 2018; *P* = 0.28). June [DOM]: [CP] was lower in 2019 vs. 2018 (5.8 ± 0.2 vs. 6.9 ± 0.2; *P* < 0.001) while September [DOM]: [CP] did not differ between years (8.1 ± 0.4 vs. 8.7 ± 0.6; *P* = 0.36).

Comparing monthly functional group composition of bison diet between 2018 and 2019, in almost all cases, patterns in June 2019 were somewhat similar to June 2018 (Table 1). For example, June protein intake from cool-season grasses was approximately 31% in both years (32.5% ± 4.4% in 2019 vs. 30.1% ± 3.9% in 2018; *P* = 0.68; Table 1). In 2019, cool-season graminoids, warm-season grasses, and legumes differed significantly from 2018. In 2019, 45.6% ± 5.1% of September dietary protein intake came from legumes (Table 1). In 2018, it was 24.6% ± 3.5% (*P* < 0.001). September dietary protein intake from cool-season graminoids was 10% lower in 2019 than 2018 (15.8 ± 2.7% vs. 26.0 ± 3.5%; *P* = 0.027). For warm-season grasses, it was 14% lower in 2019 than 2018 (30.2% ± 4.1% vs. 16.2% ± 2.9%; *P* = 0.009) (Table 1).

**Table 1:** Comparison between years of mean dietary quality and functional group abundance in diet.

Metric	Month	2018	2019	*P*
[CP]	June	93.57 ± 3.21	113.25 ± 3.33	< 0.001
	September	80.45 ± 4.73	80.61 ± 4.51	0.98
[DOM]	June	617.6 ± 3.7	630.21 ± 3.41	0.014
	September	591.71 ± 4.36	585.09 ± 4.19	0.281
[DOM]: [CP]	June	6.9 ± 0.22	5.79 ± 0.19	< 0.001
	September	8.73 ± 0.56	8.08 ± 0.4	0.361
Cool-season graminoid	June	30.07 ± 3.91	32.52 ± 4.42	0.68
	September	26.02 ± 3.45	15.8 ± 2.77	0.027
Warm-season grass	June	11.97 ± 2.08	10.29 ± 2.16	0.576
	September	30.24 ± 4.16	16.21 ± 2.94	0.009
Legume	June	32.94 ± 4.77	27.59 ± 4.54	0.418
	September	24.61 ± 3.46	45.63 ± 5.12	< 0.001
Forb	June	20.85 ± 3.72	27.65 ± 3.52	0.187
	September	16.68 ± 2.6	21.32 ± 3.46	0.277
Woody species	June	4.17 ± 1.65	1.95 ± 0.54	0.192
	September	2.45 ± 1.03	1.05 ± 0.75	0.288

Comparing relationships between [CP] with climate between the two years, although [CP] was higher on average in 2019 after standardizing for climate (*P* < 0.001), there was no difference in the relationships between [CP] and either MAT or MAP between years (*P* > 0.05 for both) (Table 2). For [DOM], 2019 [DOM] was 12.3 mg g^−1^ higher after standardizing for climate (*P* = 0.02) (Table 2). The relationships between [DOM] and MAP were similar between years (*P* = 0.22), with [DOM] decreasing at a faster rate with increasing MAT in 2019 than 2018 (− 2.6 mg g^−1^ °C^−1^ higher in 2019 than 2018; *P* = 0.05) (Table 2). After controlling for site climate, [DOM]: [CP] was 1.0 ± 0.5 lower in 2019 than 2018 (*P* = 0.02), but the relationships with climate did not differ between years (*P* > 0.1 for both) (Table 2).

**Table 2:** Model results for comparing dietary quality for June and September between 2018 and 2019.

	[CP]			[DOM]			[DOM]: [CP]		
	Estimate	SS	*P*	Estimate	SS	*P*	Estimate	SS	*P*
Intercept	97.85 ± 3.36	353,296	< 0.001	619.09 ± 4.05	14,142,475	< 0.001	6.78 ± 0.36	355.90	< 0.001
MAP.c	0.08 ± 0.01	31,490	< 0.001	0.02 ± 0.01	1285	0.147	− 0.01 ± 0	55.55	< 0.001
MAT.c	− 3.24 ± 0.68	9509	< 0.001	− 2.36 ± 0.82	5069	0.004	0.27 ± 0.07	14.12	< 0.001
Year	19.29 ± 4.4	8037	< 0.001	12.27 ± 5.3	3249	0.022	− 1.03 ± 0.47	4.84	0.029
Month (Sep)		3212	0.006		14,716	< 0.001		15.07	< 0.001
MAP.c: MAT.c	− 0.006 ± 0.0018	4667	0.001	− 0.0018 ± 0.0022	437	0.397	0.0001 ± 0.0002	0.46	0.5
MAT.c: Year	− 2.1 ± 1.08	1589	0.053	− 2.6 ± 1.3	2432	0.047	0.08 ± 0.12	0.49	0.486
MAP.c: Year	0 ± 0.01	4	0.924	0.02 ± 0.02	889	0.228	0 ± 0	2.13	0.146
Year: Month (Sep)	− 21.32 ± 6.15	5024	< 0.001	− 21.01 ± 7.41	4879	0.005	0.52 ± 0.66	0.63	0.428

Controlling for site climate, which would take into account potential differences in the distribution of sites in climate space between the years, there was no difference in the protein contribution of cool-season grasses to bison diet between years (*P* = 0.77) and no difference in relationships between climate and the protein contribution of cool-season grasses (*P* > 0.7 for both MAT and MAP) (Table 3). The same was true for warm-season grasses (Table 3). Despite significant differences in legume contribution to diet between years for the different months (*P* = 0.002), controlling for climate, legume contribution to diet was similar between years (*P* = 0.34) with no differences in the relationship between legume contribution and climate (*P* > 0.3 for both MAT and MAP) (Table 3). There were also no significant relationships with climate for forb or woody contribution to diet between years (Table 3). Despite many of the similarities between years, comparing dietary functional group composition in September between years, legumes contributed 20% more protein and warm-season grasses 14% less in 2019 than 2018.

**Table 3:** Model results for comparing functional group abundance in bison diet for June and September between 2018 and 2019.

	Cool-season Graminoid		Warm-season Grass		Legume				
	Estimate	SS	*P*	Estimate	SS	*P*	Estimate	SS	*P*
Intercept	27.78 ± 3.49	6335.47	< 0.001	12.81 ± 3.07	1739.97	< 0.001	31.15 ± 4.82	4171.5	< 0.001
MAP.c	0.0111 ± 0.0092	148	0.225	− 0.0091 ± 0.0081	127.7	0.26	0.0051 ± 0.0126	16.16	0.688
MAT.c	− 3.63 ± 0.7	2659.4	< 0.001	2.76 ± 0.62	1981.83	< 0.001	0.78 ± 0.97	63.76	0.426
Year	1.34 ± 4.56	8.58	0.77	− 0.96 ± 4.01	5.74	0.811	− 6.09 ± 6.3	93.22	0.336
Month (Sep)	− 4.33 ± 4.44	95.02	0.331	18.3 ± 3.91	2189.97	< 0.001	− 8.72 ± 6.14	201.36	0.158
MAP.c:MAT.c	0.0042 ± 0.0019	513.5	0.025	− 0.0018 ± 0.0016	123.96	0.267	0.0026 ± 0.0026	100.85	0.317
MAT.c:Year	− 0.4127 ± 1.1162	13.67	0.712	0.1801 ± 0.9823	3.36	0.855	− 0.4635 ± 1.5427	9.03	0.764
MAP.c:Year	− 0.0048 ± 0.0134	12.6	0.723	− 0.0019 ± 0.0118	2.59	0.872	0.0172 ± 0.0186	85.78	0.356
Year:Month (Sep)	− 13.57 ± 6.38	453.3	0.035	− 11.6 ± 5.61	427.45	0.04	27.93 ± 8.81	1004.58	0.002

	Forb			Woody					
	Estimate	SS	*P*	Estimate	SS	*P*			
Intercept	23.72 ± 3.54	4496.52	< 0.001	4.54 ± 1.16	1540.39	< 0.001			
MAP.c	− 0.0149 ± 0.0093	257.77	0.11	0.0078 ± 0.003	656.26	0.011			
MAT.c	0.55 ± 0.71	58.61	0.445	− 0.45 ± 0.23	372.42	0.055			
Year	7.94 ± 4.62	294.65	0.088	− 2.22 ± 1.51	216.16	0.143			
Month (Sep)	− 3.65 ± 4.51	65.66	0.419	− 1.6 ± 1.47	118.28	0.278			
MAP.c:MAT.c	− 0.0045 ± 0.0019	566.69	0.018	− 0.0005 ± 0.0006	61.89	0.433			
MAT.c:Year	0.573 ± 1.1315	25.64	0.613	0.1231 ± 0.3703	11.05	0.74			
MAP.c:Year	− 0.0056 ± 0.0136	17.12	0.68	− 0.0049 ± 0.0045	120.41	0.274			
Year:Month (Sep)	− 3.34 ± 6.46	26.78	0.606	0.59 ± 2.12	7.78	0.781			

## Discussion

Comparing the relationships between climate and dietary quality between 2018 and 2019 reveals that cooler, wetter sites generally have higher forage quality for bison than warmer, drier sites. Although not invariant, this pattern further strengthens patterns observed for cattle^8,9^ as well as those inferred from bison weight gain^7^, where bison from cool, wet climates have the highest weight gain. In contrast to previous work, these patterns did not hold for samples collected in April, where warmer sites had higher forage quality. More than likely, this is due to the earlier phenology of plants in warmer sites. Although warmer sites are more likely have a higher relative abundance of warm-season grasses than cooler sites, which can narrow phenological differences along broad temperature gradients, in April, many northern sites would have just emerged from being covered in snow while southern sites are closer to peak forage quality. This inversion is short-lived, but still would have been a driver of animal migrations assuming that bison could migrate in concert with phenological waves.

Along with the consistency in midsummer relationships between forage quality and climate between the years, functional group composition of bison diet was similar in June 2018 and June 2019. Yet, in September 2019, bison on average consumed almost twice as much protein from legumes in 2018 and correspondingly less warm- and cool-season graminoids. To better understand whether broad-scale differences in weather were behind this pattern, for each of the 45 sites measured in 2019, daily meteorological data were extracted from the Daymet surface weather database, which are provided on a 1-km grid for North America 1980-present^36^. From these data, mean August (DOY 214–244) temperature and total precipitation were calculated for 2018 and 2019. Examining August temperatures in 2018 and 2019, which presumably would influence differences in early September phenology, August temperatures were 0.1 °C higher in 2019 than 2018 (23.1 °C vs. 23.2 °C) with just 20 more mm of precipitation (109 vs. 89 mm) precipitation. Yet the similarity in average temperature masked latitudinal patterns where southern sites were approximately 1 °C warmer in 2019 and northern sites 1 °C cooler, compared to 2018 (y = 4.6 − 0.117*Latitude, *P* < 0.001). Single-site repeated measures of diet reveals similar magnitude of interannual variation in dietary composition. For example, across 3 years in a Kansas grassland, peak spring N_2_-fixing plant intake varied by 15% with a similar magnitude in variation in peak midsummer warm-season grass intake^4^. Given that, whether the differences among years are driven by differences in production between years, quality of individual species, or selection by bison is a topic for further study.

Although we have largely focused on the broad patterns here, there are still lessons to be learned from individual sites. For example, the diet of bison at Kankakee Sands in Indiana was unique. Kankakee Sands is located on a sand plain and largely consists of restored prairies dominated by warm-season grasses such as *Sorghastrum* and *Schizachyrium*. Despite the predominance of the warm-season grasses, the protein intake of bison at Kankakee Sands was dominated by *Equisetum* and *Salix*. For example, in June, > 35% of the protein intake was from *Equisetum*. *Salix* was > 85% of the protein intake in May. Overall, dietary protein concentrations were still high for the herd, reaching as much as 122 mg g^−1^ in June. Despite these high values, it appears that in a sea of low-quality grasses, bison concentrate browsing on a few species. What the dietary quality would be without *Equisetum* and *Salix*, or whether this diet is sustainable is unknown. This may be a case where management needs to begin promoting cool-season grasses and legumes to maintain high quality diets in the future in case populations of *Equisetum* and *Salix* are reduced either through over-consumption or other factors.

Overall, the predominance of eudicots for protein intake for this network of sites across 2018 and 2019 is congruent with past studies^4,37^, but still differs from assumptions of bison diet^13–17^. Part of this discrepancy could be explained by initial biases in how dietary composition was assessed, the locations of the studies, and/or the difference in focus on mass intake in earlier studies vs. protein intake in the metabarcoding studies. Still, even if eudicots had triple the protein concentration of grasses, they still would constitute approximately 40% of the mass intake. Before accepting this importance of eudicots in bison diet, there is still some further testing required. For example, the linkage of relative read abundance and relative protein intake requires scaling between chloroplast DNA concentrations of chloroplasts, chloroplast density, and protein concentrations. If some species have more copies of chloroplast DNA per chloroplast they would be “overrepresented” in the sequence counts relative to their protein contribution. Little is known about the patterns of chloroplast DNA concentrations across species and how they may change over time, but they are not constant over the maturity of leaves^38^. Setting aside the detailed molecular work on chloroplast abundance and cpDNA copies, a simple experiment that measured relationships between protein concentrations and cpDNA copies across leaves of different species would be sufficient to determine whether the generalization of scaling between the relative abundance of cpDNA among species and protein intake was a valid approximation. To date, feeding trials^39^, comparisons of diet reconstructions from microhistology and fecal DNA^40^, and isotopic estimates of the relative contribution of C_3_ and C_4_ species^4,41^ all support the validity of fecal DNA for diet reconstructions. One feeding trial did not, but that was conducted with dried hay that likely would have caused cpDNA degradation among species^42^.

In all, the research presented here illuminates one of the reasons that bison might have migrated long distances in the Great Plains, similar to the Green Wave Hypothesis^43,44^. Bison that began the spring in southern ranges would have experienced higher protein concentrations than those in northern ranges. Assuming they could have migrated fast enough to follow phenological development, this would have provided them with higher total protein intake than those that did not migrate. A migration rate of ~ 20 km d^−1^, which is similar to the migration rate of caribou^45^ and saiga^46^, would be sufficient to cover the distance between central Texas and southern Nebraska over a 2-month period. Given the interannual variation in dietary quality observed between 2018 and 2019 in June and September, it is likely that this benefit to migration would have varied among years, although more years of monitoring with data covering the entire growing season is required to more fully evaluate this question. That being said, grasslands are different now than they were even a century ago. Forage quality has declined in general^47,48^ and many northern grasslands are dominated by high-quality, introduced plant species that likely provide more protein than those they replaced. Regardless, it is a simple lesson to learn from this work that improving bison nutrition can be attained by providing access to high-protein plant species. Bison that experience protein stress during the growing season could be supplemented with protein, rangelands could be fertilized, or plants with high protein, such as N_2_-fixing species, could be promoted.

## Supplementary Information

Supplementary Information

## Data Availability

The datasets generated during and analysed for the current study are available in the Dryad repository, 10.5061/dryad.k3j9kd56m.
