# Supplementary material for: Seasonal patterns of bison diet across climate gradients in North America
Source: Sci Rep. 2021 Mar 25;11:6829. doi: 10.1038/s41598-021-86260-9 (PMC7994382; doi:10.1038/s41598-021-86260-9)
Supplement: Supplementary file 1 — Supplementary Information [file 41598_2021_86260_MOESM1_ESM.pdf]

Supplementary Information for

Seasonal patterns of bison diet across climate gradients in North America

Joseph M. Craine

Supplementary Table S1. 2019 monthly dietary quality metrics averaged (± s.e.) across all sites. Shown are crude protein concentrations ([CP]), digestible organic matter concentrations ([DOM]), and the ratio of concentrations of DOM and CP.

Month	[CP] (mg g^{-1})	[DOM] (mg g^{-1})	[DOM]:[CP]
April	82.5 ± 5.4	583.0 ± 6.1	8.1 ± 0.5
May	107.6 ± 4.4	618.1 ± 3.4	6.2 ± 0.3
June	113.2 ± 3.3	630.2 ± 3.4	5.8 ± 0.2
July	98.2 ± 4	612.4 ± 3.9	6.6 ± 0.3
August	88.1 ± 4	596.8 ± 3.8	7.3 ± 0.3
September	80.6 ± 4.5	585.1 ± 4.2	8.1 ± 0.4

Supplementary Table S2. Average (± s.e.) contributions of different functional groups to 2019 dietary protein intake for bison across sites. Data based on the 200 most abundant Exact Sequence Variants (ESVs), which were assigned a consensus taxonomy and then a function classification. Cool-season graminoids includes C_3 grasses, sedges, and *Equisetum*. Warm-season grass was derived exclusively from grasses. Legumes only included species from Fabaceae.

Month	Cool-season graminoid	Warm-season grass	Legume	Forb	Woody
April	27.6 ± 4.1	13.3 ± 2.7	33.3 ± 4.8	22.4 ± 3.7	3.5 ± 1.4
May	40.9 ± 5.0	10.4 ± 2.4	20.0 ± 4.0	24.6 ± 3.9	4.1 ± 2.1
June	32.5 ± 4.4	10.3 ± 2.2	27.6 ± 4.5	27.7 ± 3.5	2.0 ± 0.5
July	23.0 ± 3.7	10.2 ± 2.4	44.3 ± 4.8	19.6 ± 3.0	3.0 ± 2.0
August	16.6 ± 2.8	12.3 ± 2.5	55.9 ± 5.3	14.2 ± 2.9	1.0 ± 0.5
September	15.8 ± 2.8	16.2 ± 2.9	45.6 ± 5.1	21.3 ± 3.5	1.0 ± 0.7

Supplementary Table S3. Model results for dietary quality. Intercept represents April dietary quality. The interaction represents adjustments for other months.

	[CP]			[DOM]			[DOM]:[CP]		
	Estimate	SS	P	Estimate	SS	P	Estimate	SS	P
	86.3 ± 3.68	242195	<0.001	583 ± 4.38	11052230	<0.001	7.88 ± 0.28	2019.64	<0.001
MAP	0.05 ± 0.02	4549	0.001	0.00 ± 0.02	14	0.881	-0.0047 ± 0.0012	43.02	<0.001
MAT	2.51 ± 1.46	1312	0.086	2.04 ± 1.73	863	0.241	-0.16 ± 0.11	5.23	0.157
Month		38876	<0.001		77061	<0.001		200.85	<0.001
May	25.38 ± 4.77		<0.001	35.13 ± 5.67		<0.001	-1.94 ± 0.37		<0.001
Jun	31.25 ± 4.7		<0.001	47.90 ± 5.60		<0.001	-2.32 ± 0.36		<0.001
Jul	16.09 ± 4.82		<0.001	29.40 ± 5.74		<0.001	-1.48 ± 0.37		<0.001
Aug	6.5 ± 4.77		0.174	13.73 ± 5.68		0.016	-0.79 ± 0.37		0.032
Sep	-1.97 ± 4.77		0.68	1.61 ± 5.68		0.778	0.01 ± 0.37		0.977
MAP*MAT	-0.0056 ± 0.0016	5628	<0.001	-0.0000 ± 0.0019	0	0.986	0.0003 ± 0.0001	15.58	0.015
MAP*Month		1328	0.698		2741	0.496		11.36	0.497
MAP:May	0.023 ± 0.021		0.26	0.007 ± 0.025		0.781	0.001 ± 0.002		0.678
MAP:Jun	0.023 ± 0.02		0.26	0.033 ± 0.024		0.182	0.001 ± 0.002		0.577
MAP:Jul	0.030 ± 0.021		0.149	0.031 ± 0.025		0.211	0.000 ± 0.002		0.812
MAP:Aug	0.022 ± 0.021		0.285	0.032 ± 0.025		0.2	-0.001 ± 0.002	0.595	
MAP:Sep	0.033 ± 0.021		0.115	0.040 ± 0.024		0.103	-0.002 ± 0.002		0.248
MAT*Month		12502	<0.001		11686	0.003		72.74	<0.001
MAT: May	-4.52 ± 1.83		0.014	-2.11 ± 2.18		0.334	0.23 ± 0.14		0.102
MAT:Jun	-7.85 ± 1.89		<0.001	-7.55 ± 2.25		<0.001	0.45 ± 0.14		0.002
MAT:Jul	-8.48 ± 1.97		<0.001	-6.71 ± 2.34		0.005	0.57 ± 0.15		<0.001
MAT:Aug	-7.76 ± 1.92		<0.001	-6.09 ± 2.28		0.008	0.61 ± 0.15		<0.001
MAT: Sep	-7.72 ± 1.90		<0.001	-6.26 ± 2.26		0.006	0.58 ± 0.15		<0.001

Supplementary Table S4. Dietary quality model results as in Table 3, but including warm-season grass abundance.

	[CP]			[DOM]			[DOM]:[CP]		
	Estimate	SS	P	Estimate	SS	P	Estimate	SS	P
Intercept	90.33 ± 3.92	227699	<0.001	586.4 ± 4.7	9595099	<0.001	7.56 ± 0.3	1595.89	<0.001
MAP	0.04 ± 0.02	3080	0.008	-0.01 ± 0.02	165	0.606	0 ± 0	31.31	<0.001
MAT	3.5 ± 1.48	2394	0.019	2.88 ± 1.78	1621	0.106	-0.24 ± 0.11	10.91	0.038
Month		34948	<0.001		71736	<0.001		178.46	<0.001
May	24.65 ± 4.71		<0.001	34.5 ± 5.65		<0.001	-1.88 ± 0.36		<0.001
Jun	30.51 ± 4.64		<0.001	47.27 ± 5.57		<0.001	-2.26 ± 0.36		<0.001
Jul	15.31 ± 4.76		0.002	28.74 ± 5.71		<0.001	-1.42 ± 0.36		<0.001
Aug	6.38 ± 4.7		0.176	13.63 ± 5.64		0.017	-0.78 ± 0.36		0.031
Sep	-0.96 ± 4.72		0.838	2.46 ± 5.66		0.664	-0.07 ± 0.36		0.85
MAP*MAT	-0.0064 ± 0.00158	7006	<0.001	-0.00068 ± 0.0019	78	0.722	0.00036 ± 0.00012	21.71	0.004
MAP*Month		2004	0.458		3178	0.4		13.2	0.389
MAP:May	0.031 ± 0.021		0.14	0.013 ± 0.025		0.601	0.0001 ± 0.0016		0.949
MAP:Jun	0.027 ± 0.02		0.185	0.036 ± 0.024		0.141	0.0006 ± 0.0015		0.71
MAP:Jul	0.038 ± 0.021		0.067	0.038 ± 0.025		0.129	-0.001 ± 0.0016		0.524
MAP:Aug	0.026 ± 0.021		0.201	0.035 ± 0.025		0.154	-0.0012 ± 0.0016		0.458
MAP:Sep	0.04 ± 0.02		0.054	0.046 ± 0.025		0.061	-0.0024 ± 0.0016		0.129
MAT*Month		12603	<0.001		11323	0.003		69.94	<0.001
MAT: May	-5.32 ± 1.83		0.004	-2.8 ± 2.2		0.204	0.29 ± 0.14		0.949
MAT:Jun	-8.19 ± 1.87		<0.001	-7.84 ± 2.24		<0.001	0.47 ± 0.14		0.71
MAT:Jul	-8.9 ± 1.95		<0.001	-7.06 ± 2.33		0.003	0.6 ± 0.15		0.524
MAT:Aug	-7.91 ± 1.89		<0.001	-6.22 ± 2.27		0.007	0.62 ± 0.14		0.458
MAT: Sep	-7.77 ± 1.87		<0.001	-6.31 ± 2.25		0.005	0.58 ± 0.14		0.129

Supplementary Table S5. Results of model of functional group composition of bison diet. As in Table 3, the intercept represents April abundance and the interaction represents adjustments for other months.

.

	Cool-season graminoid			Warm-season grass			Legume		
	Estimate	SS	P	Estimate	SS	P	Estimate	SS	P
Intercept	27.43 ± 3.87	244.82	<0.001	15.13 ± 2.52	74.54	<0.001	30.18 ± 5.24	296.35	<0.001
MAP.c	0.01 ± 0.01	9.47	0.164	-0.01 ± 0.00	12.36	0.015	0.02 ± 0.01	48.02	0.021
MAT.c	-3.80 ± 0.54	244.94	<0.001	2.48 ± 0.35	103.95	<0.001	-0.24 ± 0.73	0.95	0.745
Month		0.3	0.803		13.64	0.256		379.18	<0.001
MonthMay	13.54 ± 5.01		0.007	-2.93 ± 3.27		0.371	-13.33 ± 6.79		0.051
MonthJun	5.45 ± 4.94		0.271	-2.93 ± 3.22		0.364	-6.53 ± 6.69		0.33
MonthJul	-4.89 ± 5.07		0.336	-2.97 ± 3.30		0.37	11.24 ± 6.87		0.103
MonthAug	-11.6 ± 5.01		0.022	-0.49 ± 3.27		0.882	22.68 ± 6.8		<0.001
MonthSep	-12.68 ± 5.01		0.012	3.48 ± 3.27		0.288	12.57 ± 6.8		0.066
MAP.c:MAT.c	0.0004 ± 0.0017	217.04	<0.001	-0.0027 ± 0.0011	13.3	0.012	0.0042 ± 0.0022	31.98	0.06
	Forb			Woody species					
	Estimate	SS	P	Estimate	SS	P			
Intercept	23.33 ± 3.72	177.1	<0.001	3.93 ± 1.54	5.032	<0.001			
MAP.c	-0.02 ± 0.01	72.73	<0.001	0.01 ± 0	4.153	0.021			
MAT.c	1.95 ± 0.52	63.99	<0.001	-0.38 ± 0.21	2.466	0.074			
Month		46.28	0.072		3.432	0.486			
MonthMay	2.17 ± 4.82		0.241	0.54 ± 1.99		0.786			
MonthJun	5.58 ± 4.75		0.56	-1.57 ± 1.96		0.424			
MonthJul	-2.85 ± 4.88		0.093	-0.54 ± 2.01		0.789			
MonthAug	-8.13 ± 4.82		<0.001	-2.48 ± 1.99		0.215			
MonthSep	-0.84 ± 4.82		0.653	-2.52 ± 1.99		0.206			
MAP.c:MAT.c	-0.0014 ± 0.0016	3.36	0.862	-0.0005 ± 0.0007	0.527	0.408			
